# The mental status of 1090 heroin addicts at entry into treatment: should depression be considered a 'dual diagnosis'?

**DOI:** 10.1186/1744-859X-6-31

**Published:** 2007-11-13

**Authors:** Icro Maremmani, Matteo Pacini, Pier Paolo Pani, Giulio Perugi, Joseph Deltito, Hagop Akiskal

**Affiliations:** 1 Vincent P. Dole Dual Diagnosis Group, 'Santa Chiara' University Hospital, Department of Psychiatry PNB, University of Pisa, Italy; 2 G. De Lisio Institute of Behavioral Sciences, Pisa, Italy; 3 AU-CNS, 'From Science to Public Policy' Association, Pietrasanta, Lucca, Italy; 4 Social-Health Integration Service, Office of Social Policies, Sardinia Health and Social Administration, Sardinia Autonomous Region, Cagliari, Italy; 5 Department of Psychiatry and Behavioral Science, New York Medical College, Valhalla, New York, USA; 6 International Mood Center, University of California at San Diego, La Jolla, and Veterans Administration Medical Center, San Diego, California, USA

## Abstract

**Background:**

Mental symptoms are common in heroin addiction and may arise from issues of addiction and withdrawal, raising doubts about the patients truly having co-morbid psychiatric diagnoses.

**Methods:**

We studied the mental status of 1090 heroin addicts (831 males and 259 females aged between 16 and 51 years) at the beginning of treatment, and its relationship to relevant demographic and clinical data through the use of standardised instruments.

**Results:**

A total of 506 (46.42%) heroin addicts showed depressive-anxious symptomatology, 421 (38.62%) had psychomotor excitement and 163 (14.95%) demonstrated a psychotic state. Patients with depressive-anxious symptomatology on the whole had a less severe addictive illness compared to those demonstrating excited and psychotic symptoms. The presence of depressive-anxious features was felt to not necessarily be indicative of the presence of a dual diagnosis.

**Conclusion:**

The presence of depressive-anxious symptomatology in the clinical presentation in heroin addicts appears to be unrelated to 'dual diagnosis'.

## Background

Drug addiction is definable as an illness [1] with core signs and symptoms (craving and relapsing behaviour) pertaining to the field of psychiatry and behavioural science [2]. Moreover, psychiatric symptoms are often prominent in states of intoxication and withdrawal, and presentations post-withdrawal are often characterised by persistent discomfort. Also, autonomous psychiatric impairment resulting from independent brain dispositions are often coupled to addiction, which makes the case one of dual diagnosis [3,4]. On the whole, psychiatric symptoms are the rule rather than the exception among heroin addicts seeking any kind of treatment, but the symptomatology that we often attribute to a comorbid mental disorder may be sometimes just be a consequence of substance use and not actually indicative of the presence of an additive psychiatric disorder.

We pose the following questions: what psychiatric mental symptom clusters occur in heroin addiction, what is the prevalence of a depressive mental cluster, and what should be considered a genuine 'dual diagnosis'?

Our study aims to provide new information about: (1) the mental status of heroin addicts who apply for treatment of their opiate addiction, and (2) The degree of association of their baseline mental status with age, sex, substance abuse history, duration of dependence and other additive, autonomous psychiatric disorder(s) not falling into the category of substance use disorder (actual 'dual diagnosis').

## Methods

### Sample

The study included 1090 heroin addicts, who had requested treatment during the years 1994–2005 at the Drug Addiction Unit of Pisa University Hospital in Pisa, Italy. All patients received a diagnosis of opioid dependence with physical dependence (according to DSM III/IIIR/IV criteria) and gave their informed consent for study participation.

The average age of the patients was 29 ± 6 years old (range 16–51). Most of the patients were male (76.2%), single (64.4%), with less than 9 years of education (70.7%), and (39.6%) were unemployed. These, and related socio-demographic characteristics, are detailed in Table 1.

**Table 1:** Socio-demographic characteristics of the sample (N = 1090)

Age, years (range):	29 ± 6 (16–51)
Sex, n (%):	
male	831 (76.2)
Civil status, n (%):	
never married	704 (64.4)
Educational level, n (%):	
low (<9 years)	771 (70.7)
Work, n (%):	
white collar	283 (26.0)
blue collar	375 (34.4)
unemployed	432 (39.6)
Income, n (%):	
poor	182 (16.7)
Living, n (%):	
in family	994 (87.1)

Males and females differed in employment rate (37.7% of males were 'blue collar workers', 37.4 were unemployed; 23.9% of females were 'blue collar workers' and 46.7% unemployed; df = 2; chi-square16.61; p = 0.0002). A total of 52.1% of males had 'blue collar worker parents and 11.8% unemployed parents, whereas 40.5% of females had 'blue collar worker' parents and 17.4% had unemployed parents (DF = 2; chi square 11.92; p = 0.002). A total of 31.5% of males were married versus 47.9% of females (DF = 1; chi square 23.07; p = 0.0000). There were no statistically significant differences between males and females with regard to age, income, education, place of birth or of residence, type of housing and whether or not they received public welfare benefits.

### Instruments

Addiction-related information was collected by means of the Drug Addiction History Rating Scale (DAH-RS, [5]) administered by a psychiatrist.

The DAH-RS is a multi-scale questionnaire comprised of the following categories: demographic data, physical health, mental health, substance abuse, treatment history, social adjustment and environmental factors, clinical characteristics as frequency of drug use, patterns of use, previous treatments, and current treatments. Items are set up so as to elicit dichotomous answers (yes/no).

For the purpose of standardised registration by means of DAH-RS, psychiatric disorders are investigated on the basis of the DSM-IV decision trees for differential diagnosis. Each decision tree starts with a set of clinical features. When one of these features is a prominent part of the presenting clinical picture, the clinician can follow the series of questions to rule in or rule out various disorders. The questions are only approximations to the diagnostic criteria and are not meant to replace them. Three decision trees have been used: 'differential diagnosis of psychotic disorders' (initial clinical features: delusions, hallucinations, disorganised speech, or grossly disorganised behaviour), 'differential diagnosis of mood disorders' (initial clinical features: depressed, elevated, expansive or irritable mood; two separate items record the presence of depression and/or any tendency towards the bipolar spectrum as testified by an elevated, expansive or irritable mood), and 'differential diagnosis of anxiety disorders' (initial clinical features: symptoms of anxiety, fear, avoidance, or increased arousal).

As for bipolar spectrum diagnoses, a history of previous hypomanic episodes, as well as temperamental characteristics, were explored using the criteria listed in the Semistructured Interview for Depression (SID) [6]. All information was gathered from the patient and at least one close relative (usually parents/siblings); in addition, all available clinical records were carefully examined. Inquiry on temperamental attributes was made about the habitual state of the patient during periods free of affective episodes; this was from both the patient and significant others. Our operational criteria for affective temperaments were the University of Tennessee [7] modifications of the Schneiderian descriptions [8]. The SID, developed as part of the Pisa-Memphis (now San Diego) collaborative study on affective disorders, has been used with over 2500 patients at the time of writing: its reliability for diagnostic assessment of patients and their temperaments has been documented elsewhere [9,10].

### Statistical analyses

To describe the mental status, we used the following DAH-RS items: awareness of illness, consciousness disturbances, memory deficits, anxiety states, depression, sleep disturbances, eating disturbances, excitement, violence, suicidality, delusions, and hallucinations.

A factor analysis was performed on these DAH-RS items for 1090 patients in order to identify possible composite dimensions. The initial factors were extracted by means of principal component analysis (type 2) and then rotated according to varimax criteria in order to achieve a simple structure. This simplification is equivalent to maximising the variance of the squared loading in each column. The criterion used to select the number of factors was an eigenvalue greater than 1. Item loadings with absolute values greater than 0.4 were used to describe the factors. This procedure makes it possible to minimise the correlations between the factors, so allowing their optimisation as classificatory tools for each subject. The factorial scores were then standardised as z-scores to facilitate the comparisons of scores among the factorial measures. All the subjects were then grouped into different subtypes on the basis of the highest z-scores obtained for each factor (dominant factor). We compared clinical features among the various dominant factor groups by means of one-way ANOVA followed by the Student-Neuman-Keultz F-test for metrical variables and chi-square test for category variables.

Statistical analyses were carried out using the SPSS package (version 4.0 by SPSS Inc). As this is an exploratory study, statistical tests were considered significant at the p < 0.05 level.

## Results

Only 376 (34.5%) patients showed awareness of their illness, which means that this group conceptualise themselves as having a self-maintaining disease caused by previous exposure to heroin, and are in need of some long-term relapse prevention. A total of 499 (45.8%) reported depressed mood, and 437 (40.1%) report preoccupation with somatic functions or experience of spontaneous anxiety. Sleep disorders were reported by 374 subjects (34.3%), and (hypo)manic or mixed excitement was displayed in a prominent way by 262 (24.0%) patients at time of evaluation; 230 (21.1%) felt or showed aggressive behaviours and/or had been violent recently; 97 (8.9%) had engaged in self-injurious acts. Appetite was increased or decreased in 173 (15.9%) subjects. Memory disturbance occured in 139 (12.8%) subjects. The least featured symptoms were delusions (n = 76; 7.0%), altered states of consciousness (n = 70; 6.4%) and hallucinations (n = 51; 4.7%).

Significant gender-related differences for psychic impairment at treatment entrance were revealed by univariate analysis (Table 2). Females were more likely to display eating disturbances, states of anxiety, depression, violence, psychomotor excitement, suicidal thoughts and behaviours, and delusions. On the whole, women tended to have a higher level of psychic impairment at treatment initiation.

**Table 2:** Mental status of 1090 heroin addicts at beginning of the treatment

	Total n (%)	Males n = 831	Females n = 259	Chi-square	p Value
Awareness of illness	376 (34.5)	295 (35.5)	81 (31.3)	1.56	0.21
Consciousness disturbance	70 (6.4)	51 (6.1)	19 (7.3)	0.47	0.49
Memory disorders	139 (12.8)	98 (11.8)	41 (15.8)	2.89	0.08
Anxiety state	437 (40.1)	315 (37.9)	122 (47.1)	6.95	0.008
Depression	499 (45.8)	359 (43.2)	140 (54.1)	9.37	0.002
Sleep disturbances	374 (34.3)	274 (33.0)	100 (38.6)	2.78	0.09
Eating disturbances	173 (15.9)	104 (12.5)	69 (26.6)	29.50	<0.001
Psychomotor excitement	262 (24.0)	186 (22.4)	76 (29.3)	5.24	0.02
Violence	230 (21.1)	159 (19.1)	71 (27.4)	8.13	0.004
Suicidality	97 (8.9)	64 (7.7)	33 (12.7)	6.18	0.012
Delusions	76 (7.0)	50 (6.0)	26 (10.0)	4.92	0.02
Hallucinations	51 (4.7)	34 (4.1)	17 (6.6)	2.70	0.09

Principal component factor analysis of the selected 12 items of DAH-RS yielded a three-factor solution, with a loading greater than 0.40 (Table 3). The first factor reflects a 'depressive-anxious' dimension (illness awareness, anxiety state, depressed mood, sleep and eating disturbances), which accounted for 31.1% of the variance. The second factor, accounting for 11.9% of the variance, reflects a psychomotor excitement dimension (hypomanic/manic or mixed state, aggressiveness and violence, suicidality). The third factor, which reflects a 'psychotic state' dimension, included memory deficits, altered consciousness, delusions, and hallucinations, accounted for 10.3% of the total variance.

**Table 3:** Factors analysis (PCA method and varimax rotation)

	F1	F2	F3
Awareness of illness	0.47		
Consciousness disturbance			0.58
Memory deficits			0.40
Anxiety state	0.71		
Depression	0.73		
Sleep disturbances	0.74		
Eating disturbances	0.57		
Psychomotor excitement		0.91	
Violence		0.91	
Suicidality		0.48	
Delusions			0.79
Hallucinations			0.74
% Variance	31.1	11.9	10.3

On the basis of the highest z-scores obtained for each factor (dominant factor) we clustered all the subjects into three groups. The dominant 'depressive-anxious' group comprised 506 subjects (46.4%), the dominant 'psychomotor excitement' group 421 (38.6%), and the dominant 'psychotic state' group 163 (15.0%). Comparison of these groups (Table 4) revealed significant differences.

**Table 4:** Differential characteristics of heroin addicts with different psychopathological syndromes at the initiation of their treatment

	Depressive-anxious (Group 1), n = 506	Psychomotor excitement (group 2), n = 421	Psychotic state (group 3), n = 163			
	**M ± (sd)**	**Mean (sd)**	**Mean (sd)**	**T/F**	**p Value**	**Contrast between groups**

Age	29.30 ± 6.7	29.47 ± 6.2	29.46 ± 5.8	0.92	0.91	
Age of first contact	18.49 ± 3.7	18.78 ± 3.8	17.80 ± 3.9	3.88	0.02	2 ≠ 3
Age of onset of dependence	20.75 ± 4.1	21.46 ± 4.9	19.90 ± 4.3	7.67	<0.001	2 ≠ 3
Duration of dependence	85.49 ± 69.9	79.38 ± 64.8	90.80 ± 70.3	1.90	0.14	
Age fist therapeutic contact	25.60 ± 5.2	25.16 ± 5.2	24.64 ± 5.2	2.25	0.15	
No of physical complaints	1.13 ± 0.9	1.57 ± 0.8	2.06 ± 0.9	29.80	<0.001	1 ≠ 2 ≠ 3
No of abused substances	2.29 ± 1.7	3.32 ± 1.6	3.75 ± 1.6	67.78	<0.001	1 ≠ 2 ≠ 3
No of past treatments	1.07 ± 0.9	1.08 ± 0.8	2.10 ± 0.9	52.07	<0.001	1 ≠ 2 = 3

	**n (%)**	**n (%)**	**n (%)**	**Chi-square**	**p Value**	**Contrast**

Gender (Males)	392 (77.5)	317 (75.3)	122 (74.8)	0.80	0.66	
Civil Status (single)	329 (65.0)	282 (67.0)	93 (57.1)	5.14	0.07	
Educational level (low)	390 (77.1)	267 (63.4)	114 (69.9)	20.70	0.00003	1 ≠ 2 = 3
Income (poor)	59 (11.7)	88 (20.9)	25 (21.5)	17.25	0.00018	1 ≠ 2 = 3
Heroin intake (daily)	417 (82.4)	310 (73.6)	125 (76.7)	10.61	0.0049	1 ≠ 2 = 3
Modality of use (stables)	361 (71.3)	231 (54.9)	69 (42.3)	53.05	<0.00001	1 ≠ 2 ≠ 3
Phase (revolving door)	402 (79.4)	306 (72.7)	117 (71.8)	7.30	0.02	1 ≠ 2 = 3
Concurrent use of alcohol	116 (22.9)	168 (39.9)	85 (52.1)	58.23	<0.00001	1 ≠ 2 ≠ 3
Concurrent use of BDZ	85 (16.1)	151 (35.9)	65 (39.9)	56.2	<0.00001	1 ≠ 2 ≠ 3
Concurrent use of Cocaine	177 (35.0)	208 (49.4)	101 (62.0)	42.7	<0.00001	1 ≠ 2 ≠ 3
Concurrent use of THC	250 (49.4)	306 (72.7)	129 (79.1)	75.1	<0.00001	1 ≠ 2 ≠ 3
Job issues	299 (59.1)	255 (60.6)	105 (64.4)	1.40	0.48	
Household issues	262 (51.8)	198 (47.0)	100 (61.3)	9.7	0.007	2 ≠ 3 = 1
Sexual issues	270 (53.4)	162 (38.5)	64 (39.3)	23.52	0.0001	1 ≠ 2 ≠ 3
Social\leisure issues	298 (58.9)	205 (48.7)	97 (59.5)	11.20	0.003	3 = 1 ≠ 2
Legal issues	158 (31.2)	147 (34.9)	81 (49.7)	18.45	0.0001	2 = 1 ≠ 3
Double diagnosis (presence)	65 (12.8)	377 (89.5)	138 (84.7)	619.18	<0.0001	1 ≠ 2 ≠ 3

Depressive-anxious heroin addicts had milder somatic impairment, abused fewer (other) substances, and underwent fewer types of treatment attempts. Their educational level was on the average lower, their economic conditions were better, heroin use was daily or less frequent for most, rather than 'more times a day' as in other groups. The addictive mode (i.e. the kind of lifestyle while using heroin) was typically the 'stable' one, that is maintaining productivity and not engaging in street crime despite major individual and relational impairment, common to all DAH-RS profiles. Nevertheless, the majority in this group had undergone a series of relapses and repeatedly failed to maintain abstinence (so-called 'revolving door stage'). The prevalence of concurrent use of alcohol, unprescribed benzodiazepines, cocaine and cannabis is lower. Social adjustment is impaired to a greater extent in dominant 'psychomotor excitement' patients. Lastly, dominant 'depressive-anxious' patients were less likely to be rated as having a 'dual diagnosis' by a more thorough psychiatric evaluation by means of DSM and SID. In other words, it can be stated that the global weight of addiction-related impairment is lower for dominant 'depressive-anxious', in comparison with dominant 'psychomotor excitement' or 'psychotic state' peers. Baseline conditions of dominant psychomotor excitement and psychosis are more likely to correspond to the otherwise assessed presence of an additive and autonomous psychiatric disorder (dual diagnosis).

## Discussion

A current major depressive episode has been reported in 16–34% of patients seeking treatment [11-16], this percentage being higher than among those out of any treatment [17]. Females are more likely to have a current major depressive episode [17]. In our study the percentage of patients who were depressed when entering treatment (about 46%) is higher than what reported by other authors, and in particular about 50% of female patients are undergoing an episode of major depression. A similar percentage of patients (46%) fell into the dominant 'depressive-anxious' group, including depressed mood together with anxiety states, sleep disturbance and altered appetite. In our study, the depressive-anxious dimension, built in as a factor, is also the most represented (31.1% of total variance). However, the depressive-anxious factor isn't related to the severity of addiction history, nor it is strongly linked to the presence of a dual diagnosis.

The 'psychomotor excitement' dimension, also accounting for aggressiveness and self-injurious behaviour, is prominent for 38.62% of subjects. On clinical and psychopathological grounds, psychomotor excitement is usually related to the presence of dual diagnosis, with special regard to bipolar disorder. Of note, the prevalence of such a factor in our sample is far higher than previous literature rates of manic/hypomanic and mixed states, ranging from 0.1% [16,18] to 5.5% [19,20], but in the sub-sample recruited in our university psychiatric setting rates of bipolar I and II were even higher (51–55%) [21,22]. Additionally, in our study dominant psychomotor excitement patients were more likely to be assessed as having a dual diagnosis than are dominant depressive-anxious patients. Subjects who present prominently as psychotic upon entering treatment are far more uncommon (14.9%) with regard to a factor accounting for delusion (7.0%) hallucinations (4.7%), memory disturbance (12.8%), or altered consciousness (6.4%). Other authors agreed, reporting low rates for psychosis among heroin addicts [23-25], yet we found even lower rates. These subjects are also more likely to be affected by a dual diagnosis in comparison with depressive-anxious subjects. Lastly, in our sample, heroin-dependent patients tend to have poor illness awareness regardless of presence a true dual diagnosis.

## Conclusion

Our data show that depressive features are the most frequent psychiatric symptoms among heroin addicts looking for treatment, and part of the most highly represented clinical state (depressive-anxious). However, the depressive features of heroin addicts are associated with anxiety rather than with suicidality, and a dominant depressive-anxious state is linked to a lower severity of drug addiction history. Conversely, psychomotor excitement and psychosis predict the presence of polysubstance abuse and an actual dual diagnosis. Therefore, it is very important for clinicians to be able to identify major as well as minor psychomotor excitement and psychotic symptoms in heroin addicts presenting for treatment, because it is likely that these patients are affected by another independent mental disorder (dual diagnosis) that deserves specific clinical attention and treatment. By contrast, the presence of depressive features in the clinical presentation of heroin addicts appears to be an unreliable indicator of general psychiatric severity and appears to be a common comorbid condition of the average addict, also developing at lower levels of addiction severity, along with the early course of the addictive disease. In conclusion, our data indicate something opposite to the general trend to identify dual diagnosis from baseline depression patients and ascribe psychomotor excitement or psychosis to intoxication from psychoactive substances: depressive-anxious symptoms are more likely to be parallel to addiction as a non-specific form of psychic disturbance, whereas psychomotor excitement and psychotic ones are likely to have dual diagnosis.

## Competing interests

The author(s) declare that they have no competing interests.

## Authors' contributions

All the authors contributed equally to this work
